# Powerful terahertz waves from long-wavelength infrared laser filaments

**DOI:** 10.1038/s41377-020-00423-3

**Published:** 2020-11-12

**Authors:** Vladimir Yu. Fedorov, Stelios Tzortzakis

**Affiliations:** 1 Science Program, Texas A&M University at Qatar, P.O. Box 23874, Doha, Qatar; 2 P.N. Lebedev Physical Institute of the Russian Academy of Sciences, 53 Leninskiy Prospekt, Moscow, 119991 Russia; 3 Institute of Electronic Structure and Laser (IESL), Foundation for Research and Technology-Hellas (FORTH), P.O. Box 1527, Heraklion, GR-71110 Greece; 4 Department of Materials Science and Technology, University of Crete, Heraklion, GR-71003 Greece

**Keywords:** Terahertz optics, Nonlinear optics

## Abstract

Strong terahertz (THz) electric and magnetic transients open up new horizons in science and applications. We review the most promising way of achieving sub-cycle THz pulses with extreme field strengths. During the nonlinear propagation of two-color mid-infrared and far-infrared ultrashort laser pulses, long, and thick plasma strings are produced, where strong photocurrents result in intense THz transients. The corresponding THz electric and magnetic field strengths can potentially reach the gigavolt per centimeter and kilotesla levels, respectively. The intensities of these THz fields enable extreme nonlinear optics and relativistic physics. We offer a comprehensive review, starting from the microscopic physical processes of light-matter interactions with mid-infrared and far-infrared ultrashort laser pulses, the theoretical and numerical advances in the nonlinear propagation of these laser fields, and the most important experimental demonstrations to date.

## Terahertz light: taking plasma filaments on a power trip

An experimental technology that can reduce the cost and complexity of generating powerful terahertz waves could benefit fields such as wireless communication. Stelios Tzortzakis from the IESL-FORTH in Heraklion, Greece, and colleagues review efforts to understand and optimize a process that uses ultrafast laser pulses to ionize gases into thin filament plasma antennas. Careful laser frequency mixing then enables the filaments to emit terahertz radiation, which is broadband and powerful enough to pass through buildings and walls without risk to human health. Theoretical and experimental studies have highlighted the importance of using laser sources with specific wavelengths to enhance the power conversion efficiency of terahertz radiation and enable long-range propagation in air. Improved schemes for controlling frequency mixing are projected to offer order-of-magnitude increases in terahertz power output compared to current approaches.

## Introduction

The terahertz (THz) frequency range is the last lacuna in the electromagnetic spectrum, where we have no efficient sources of radiation. Being usually understood as the region of frequencies from 0.1 to 10 THz or, equivalently, of wavelengths from 3 mm to 30 μm, the THz part of the spectrum is squeezed between the domains of high-frequency electronics and photonics. While a large number of powerful microwave sources exist in high-frequency electronics and high-energy lasers cover the needs in photonics, there is currently no easy, direct way to generate THz fields of any comparable strength.

The THz frequency range draws attention for a number of reasons. First, THz waves penetrate almost losslessly through a large number of different materials, such as plastics, fabrics, concrete, wood, and paper. However, unlike X-rays, the energy of THz photons, being on the order of several meV, is too low to directly disrupt any chemical bonds or cause electronic transitions in a medium and thereby damage it. As a result, THz radiation becomes very attractive for various applications related to imaging and diagnostics in areas such as medicine, industrial quality control, food inspection, or artwork examination^1–4^. Moreover, a large number of processes at the microscopic level occur with characteristic times corresponding to terahertz frequencies, for example, rotations and vibrations in large molecules (nucleic acids, proteins, synthetic polymers, etc.), lattice vibrations and free carrier motion in solids. Therefore, THz waves serve an important role in fundamental studies of matter and find their applications in spectroscopy and the control of materials, including ultrafast electric-field and magnetic-field switching, being much faster than in conventional electronics^5,6^. In addition, there is a whole set of applications that directly rely on strong, high-energy THz fields, for example, table-top THz electron acceleration^7–9^ or THz enhancement of attosecond pulse generation^10,11^.

Despite the existing need for strong THz radiation, the list of available powerful THz sources is quite restricted^12^. Currently, there are two major techniques that allow one to generate intense THz fields on a table top: optical rectification in electro-optic crystals^13–15^ and two-color filamentation in gases and liquids^16–20^. In THz generation by optical rectification, a crystal with a second-order nonlinearity is pumped by an ultrashort laser pulse; the difference frequency mixing of various spectral components in the wide pulse spectrum produces a beating polarization that gives rise to the emission of radiation in the THz spectral region. In turn, two-color filamentation is based on the ionization of a medium by an ultrashort laser pulse consisting of fundamental radiation and its second harmonic. Then, free electrons of the generated plasma oscillate in the driving laser field and emit electromagnetic waves. By a proper choice of the phase between the fundamental and second harmonic pulses, it is possible to break the field symmetry and force the free electrons to produce a residual photocurrent oscillating at THz frequencies^21^.

Among the two above techniques, optical rectification allows one to generate THz pulses with higher energy, up to 0.9 mJ^14^, and higher THz conversion efficiency (the ratio of the THz energy to the energy of the input two-color laser pulse), up to 3.7%^22^. Unfortunately, inevitable optical damage of nonlinear crystals by high laser intensities makes further step ups in THz energy very troublesome. In addition, THz pulses generated by optical rectification are spectrally quite narrow (the spectral width is below 5 THz) and, as a result, are quite long (the corresponding pulse durations are approximately several picoseconds). On the other hand, common two-color filamentation with near-infrared laser pulses offers less energetic THz radiation (up to 30 μJ in gases^18^ and up to 80 μJ in liquids^20^) with less THz conversion efficiency, which is usually close to 0.01%. Nevertheless, since the gas and liquid media recover in-between laser shots, the optical damage issue becomes irrelevant. Moreover, two-color filamentation allows one to generate ultrashort single-cycle THz pulses with femtosecond duration and spectral bandwidths exceeding 50 THz^23,24^. Therefore, despite the lower energies, these THz pulses can have a very high intensity. Furthermore, two-color filamentation makes it possible to generate THz radiation remotely, thereby avoiding challenges such as the high absorption of THz waves in atmospheric water vapor^25–28^.

In addition, the THz radiation generated by optical rectification and two-color filamentation has different emission patterns. Unlike optical rectification, where the spatial profile of the generated THz radiation repeats the shape of the pump laser beam, the THz radiation produced by two-color filamentation is emitted as a cone whose angle depends on the thickness of the plasma channel and its length^29–31^.

**Fig. 1:**
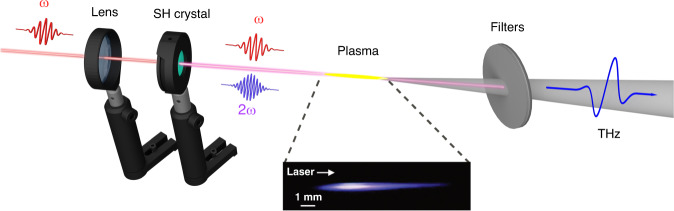
Typical setup for THz generation by two-color filamentation. The fundamental component of a laser pulse is focused through a nonlinear second harmonic (SH) crystal into ambient air. The second harmonic generated in the nonlinear crystal mixes with the fundamental pulse and provides the desired field symmetry breaking (the phase between the *ω* and 2*ω* fields is controlled by the crystal position along the optical axis). The plasma channel generated close to the lens focus emits THz radiation, which is then filtered out by a set of longpass filters (in most cases, high-resistivity silicon wafers)

Figure 1 shows a typical experimental setup for THz generation by two-color filamentation.

Due to the prevalence of commercially available high-power laser systems operating at near-infrared wavelengths, most experiments on both optical rectification and two-color filamentation were conducted using near-infrared laser pulses. While the choice of the pump wavelength for optical rectification is mainly dictated by the phase-matching properties of the available nonlinear crystals, the wavelength of the laser source for two-color filamentation can be chosen more freely. Taking into account that many parameters governing the physics of ionization and the motion of electrons in the laser field depend on its wavelength, we can assume that certain laser wavelengths will be beneficial for two-color filamentation. In particular, since the ponderomotive energy of free electrons under the action of a laser field increases as the square of the field’s wavelength, it is reasonable to expect that laser sources operating at longer wavelengths should produce stronger photocurrents and, consequently, more energetic THz radiation. However, to produce filaments with long and dense plasma channels favorable for THz generation, the peak power of a laser pulse should exceed the critical power for Kerr self-focusing (otherwise, the dense enough plasma can be produced only by tightly focused laser pulses and only in the vicinity of the focal spot). In turn, since the critical power for Kerr self-focusing also scales as the wavelength squared, until recently, the experimental investigation of two-color filamentation at wavelengths from mid-infrared and far-infrared parts of the spectrum was almost prohibitive because of the demanding optical power requirement.

Everything changed with the advent of a new generation of optical parametric chirped-pulse amplifiers (OPCPAs) capable of delivering high-peak-power sub-100 fs pulses at a central wavelength of 3.9 μm^32–34^. Recent advances in the development of high-power pulsed CO_2_ lasers are also very encouraging^35,36^. With these newly appeared laser systems, the studies of laser filamentation in gases have been extended to the mid-infrared and far-infrared spectral ranges. The first experiments on long-wavelength filamentation revealed a number of unique regimes of nonlinear spatiotemporal wave dynamics and demonstrated many unusual aspects in the nonlinear-optical response of gaseous media to mid-infrared and far-infrared laser radiation^37,38^. One can easily expect that these peculiar laser-matter interactions will also be reflected in the generation of THz radiation by the two-color filamentation of long-wavelength laser pulses.

Here, we review the recent experimental and theoretical advances in the interaction of long-wavelength laser radiation with gases in the context of two-color filamentation, which show great promise as sources of extremely powerful THz waves. We carefully analyze how the complex nonlinear-optical response of gaseous media on mid-infrared and far-infrared radiation can affect the process of THz generation and what the prerequisites are for stronger and more powerful THz fields. The theoretical analysis is completed with a review of the current state of research on the nonlinear propagation of mid-infrared and far-infrared two-color filaments.

## Medium response

### Ionization and free electrons

Ionization plays a central role in the process of THz generation by two-color laser filamentation. Following the photocurrent model^21^, THz radiation originates from the current *J* of free electrons oscillating under the action of an electric field with broken symmetry. In this framework, the amplitude of the emitted THz pulses is proportional to the first time derivative of the electron current $\partial J/\partial t$. Since the magnitude of *J* depends linearly on the electron velocity *v*, the energy of the generated THz radiation should be proportional to *v*^2^ and thus to the kinetic energy of the electrons acquired within the electric field.

A classical nonrelativistic equation of motion for an electron driven by a field *E* can be written as $dv(t)/dt = eE(t)/m$, where *e* and *m* are the charge and mass of the electron, respectively. According to this equation, under the action of an electric field $E(t) = A{\mathrm{cos}}(\omega t)$, the electron gains velocity $v(t) = eA{\mathrm{sin}}(\omega t)/m\omega$. The corresponding average kinetic energy is

1 $$U_p = < \frac{{mv^2}}{2} > = \frac{{e^2A^2}}{{4m\omega ^2}} \propto I\lambda ^2$$

where *A*, *I*, and *λ* are the field amplitude, intensity, and wavelength, respectively. This energy, known as ponderomotive or quiver energy, is one of the basic parameters governing strong-field physics.

Thus, the most straightforward way to obtain more energetic THz radiation is to increase the ponderomotive energy *U*_p_ of plasma electrons. According to Eq. (1), this can be accomplished either by increasing the pulse intensity *I* or its wavelength *λ*. Obviously, the wavelength is a more effective control knob because *U*_p_ depends on *λ* quadratically but only linearly on *I*. Moreover, the maximum laser intensity during filamentation is restricted by the effect of intensity clamping^39^. Since the energy of THz pulses linearly depends on *U*_p_, it should also scale quadratically with the laser wavelength. The latter conclusion is the main motivation for increasing the laser wavelength in studies of THz sources driven by laser filamentation.

**Fig. 2:**
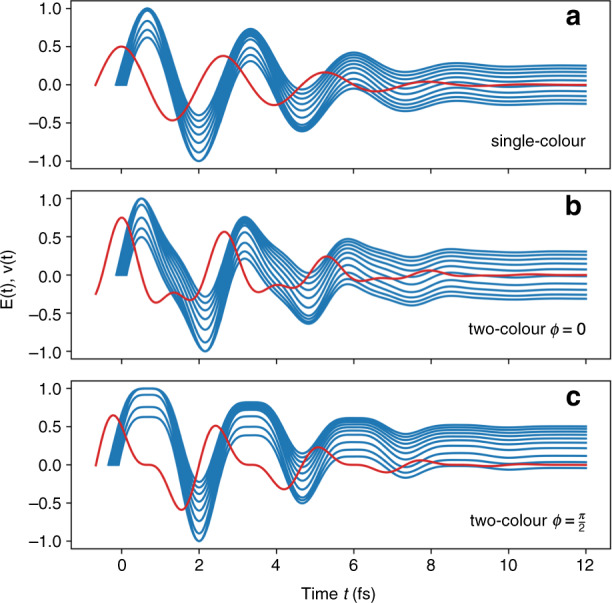
Mechanism of THz generation by two-color filamentation. Electron velocities *v*(*t*) (blue lines) for several electrons released at different time instances close to the peak of the driving field *E*(*t*) (red lines). **a** Single-color laser field $E(t) = A(t)\cos (\omega _0t)$. **b**, **c** Two-color laser field $E(t) = A(t)\cos (\omega _0t) + [A(t)/2]\cos (2\omega _0t + \phi )$ with **b** *ϕ* = 0 and **c** *ϕ* = *π*/2. The amplitude $A(t) = \exp ( - t^2/\tau _0^2)$, where *τ*_0_ = 5 fs. The central frequency *ω*_0_ corresponds to a wavelength of 800 nm

Another important aspect of the ionization process involves the Keldysh parameter *γ*, which can be expressed through the ponderomotive energy *U*_p_ as

2 $$\gamma = \sqrt {\frac{{U_{\mathrm{i}}}}{{2U_{\mathrm{p}}}}} \propto \frac{1}{{\sqrt I \lambda }}$$

where *U*_i_ is the ionization energy of an atom or a molecule. The value of *γ* distinguishes the tunneling (*γ* ≪ 1) and multiphoton (*γ* ≫ 1) regimes of ionization^40^. For an atom or molecule at constant intensity, *γ* scales as 1/*λ*. Therefore, longer wavelengths drive ionization further into the tunneling regime. In turn, an extremely fast, exponential dependence of the tunnel ionization rate on the field amplitude^40^ implies that photoelectrons are released in a narrow range of time close to the peaks of the field. As a result, the maximum ponderomotive energy that an electron can gain will depend on the particular phase of the field at the moment of electron release. Figure 2 shows the temporal evolution of several electron velocities *v*(*t*) within the driving laser field for the case of single-color and two-color laser pulses with different phases. In Fig. 2a, one can see that at the end of a single-color laser pulse, the final velocities of individual electrons become uniformly distributed around zero, resulting in a zero average photocurrent. The situation is similar in the case of two-color laser pulses with zero phase difference *ϕ* between the fundamental and the second harmonic components (see Fig. 2b). However, when *ϕ* = *π*/2 (see Fig. 2c), most of the electrons at the end of the laser pulse acquire final velocities of the same sign, giving rise to a nonzero residual photocurrent, which is responsible for THz generation^21^. Thus, the concept of sub-cycle tunnel ionization in a time-varying field becomes central for studies of mid-infrared and far-infrared two-color filamentation. In contrast, in the limit of short wavelengths, corresponding to multiphoton ionization (*γ* ≫ 1), the effect of the field phase on the ionization process vanishes. In this case, the ionization probability (measured in inverse seconds) is much less than an optical cycle, and many field periods should pass to release a single free electron. As a result, the ionization rate becomes dependent on the field envelope or, equivalently, on the cycle-averaged intensity rather than the instantaneous field strength. Therefore, the asymmetry introduced by two-color fields smooths out, leading to less efficient THz generation. However, effective THz generation in the multiphoton regime can still be achieved if one uses a laser field with two frequencies of noninteger ratio (i.e., *ω* and 2*ω* + δ*ω*), which result in fast beatings of the pulse envelope^41^.

To date, a large amount of data on the ionization of gases by mid-infrared laser pulses has been obtained in experiments on high-harmonics generation^42^. These experiments confirmed that the kinetic energy of photoelectrons indeed scales as *λ*^2^. Moreover, they demonstrated that with an increase in the laser wavelength, the photoelectron energy spectrum evolves towards the structure predicted by the semi-classical theory (known as a simple man model)—a spectrum clipped by 2*U*_p_ followed by a plateau descending to the 10*U*_p_ cutoff caused by photoelectron rescattering and re-acceleration by the field^43^. Thus, the physics of ionization and motion of photoelectrons with increasing laser wavelength tends closer to the classical scenario.

**Fig. 3:**
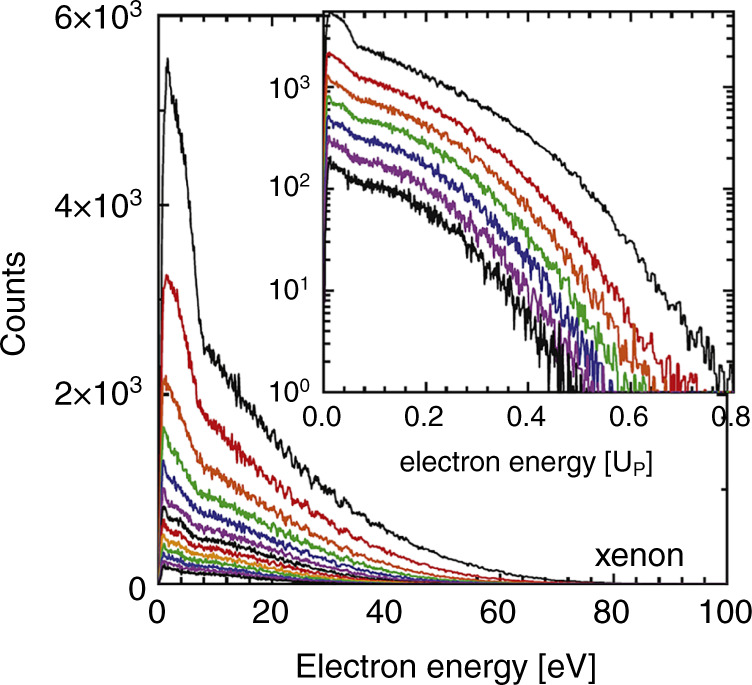
Low-energy photoelectron spectrum in the tunneling regime. Dependence of the photoelectron spectra of xenon on the intensity of 3.6 μm, 140 fs laser pulses. The laser intensity used to generate the spectra (presented in different colors) was varied in steps of 0.025*I*_0_, where *I*_0_ = 0.65 × 10^14^ W cm^−2^ is the maximum laser intensity. The spectra exhibit a spike-like enhancement for electron energies ${\boldsymbol{E}} \lesssim 10\,{\mathrm{eV}}$, known as the low-energy structure. The inset displays the spectrum on a semi-logarithmic scale for electron energies in units of ponderomotive energy *U*_p_. Reprinted from ref. ^43^ under the terms of the Creative Commons Attribution 3.0 licence

Apart from the confirmation of theoretical expectations, experiments on high harmonic generation with mid-infrared laser pulses led to the discovery of a previously unknown feature in the energy distribution of photoelectrons. It was found that the lowest energy part of the photoelectron energy spectrum has an unexpected peak, the so-called low-energy structure, which becomes prominent only when using long-wavelength laser pulses^43–45^ (see Fig. 3). Interestingly, it appears that these low-energy electrons are tightly connected to the emitted THz waves and can shed light on the microscopic mechanisms of THz generation^46^.

**Fig. 4:**
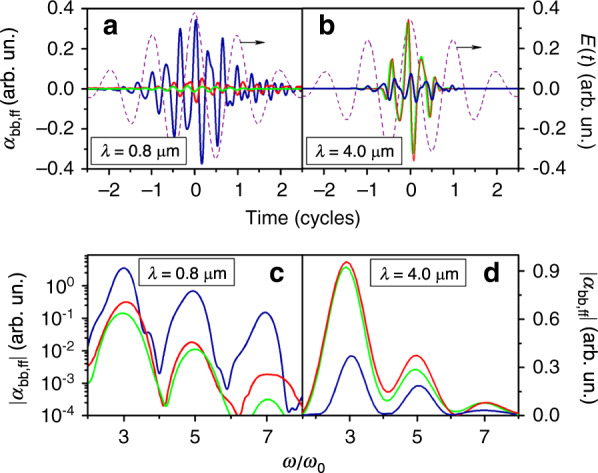
Comparison of radiation amplitudes of bound and free electrons at different laser wavelengths. Time-resolved radiation amplitudes *a*_bb_ (blue) and *a*_ff_ (red) of bound-state and free electrons **a**, **b** and their spectra **c**, **d** for a laser pulse (purple dashed line) with an intensity of 110 TW/cm^2^ and wavelengths of 0.8 μm **a**, **c** and 4 μm **b**, **d**. The results of calculations using the semi-classical model for free electrons are indicated by the green line. Reprinted from ref. ^47^ with permission from the American Physical Society

Another distinguishable feature of ionization driven by long-wavelength laser pulses is related to the increasing role of free electrons in the overall nonlinear response of a medium. The solution of the Schrödinger equation for a generic hydrogen quantum system shows that for high-intensity long-wavelength fields, electrons released after ionization can travel far away from the atomic core, acquiring high energy within the long field period^47^. In this regime, the electron wave function is no longer tightly localized around the atomic core, and a significant fraction of electrons undergoing ionization do not recombine back to bound states, building up the continuum population in a stepwise fashion after each field half-cycle. The steps in the continuum population, synchronized with the field half-cycle, translate into harmonics whose intensity is much higher than the intensity of harmonics emitted by bound-state electrons (see Fig. 4). As a result, for high-intensity long-wavelength pulses, free-state electrons dominate over bound electrons in the overall nonlinear response. Thus, since for longer laser wavelengths, the behavior of free electrons tends to be more classical, the use of simple semi-classical models for studies of mid-infrared and far-infrared two-color filamentation seems appropriate.

To complete the semi-classical description of ionization for long-wavelength laser pulses, we have to indicate the way to calculate the corresponding ionization rate. Recently, Lai et al.^48^ experimentally studied the ionization yield versus laser intensity for various atomic targets irradiated by laser pulses with different polarizations and wavelengths ranging from the visible to the mid-infrared (0.4–4 μm) in both the multiphoton and tunneling regimes. The experimental findings were compared to the ionization rate calculated by the Perelomov-Popov-Terent’ev (PPT) formula^49^. It was shown that the PPT formula, corrected by a generalized Coulomb prefactor^50^, agrees well with all of the experimental data, including the ionization yield ratio between circular and linear laser polarizations.

The estimations of classical electron trajectories under the action of strong mid-infrared and far-infrared laser fields show that tunneling electrons can travel away from the parent atom or molecule over distances comparable to a collisional mean free path in gases^38^. Consequently, an electron released from a parent atom or molecule can interact with many other atoms or molecules located nearby. In particular, on the way from its birthplace, such an electron can acquire enough energy to ionize neighboring atoms or molecules and give rise to significant avalanche ionization. Thus, compared to near-infrared laser fields, the theoretical description of laser-plasma interactions with mid-infrared and far-infrared laser pulses requires paying more attention to many-body effects.

For example, recent theoretical studies suggest that many-body effects in gases can lead to an additional ionization channel through excitation-induced dephasing (EID), which manifests itself at low laser intensities^51–54^. It was found that the following semi-phenomenological formula for the ionization rate provides a good fit to the full many-body quantum theory of EID^38,54^:

3 $$R(E) = C_{{\mathrm{MBI}}}E^4(t)\sqrt {\frac{{E^2(t) + s}}{{E^2(t)}}}$$

with *C*_MBI_ = 2.84 × 10^−7^ (m^−3^s^−1^)(m^4^V^−4^) and *s* = 4.6 × 10^18^ V^2^m^−2^. According to Eq. (3), the additional ionization yield proposed by EID calculations scales quadratically with the laser intensity *I*, in strong contrast to the much higher intensity powers predicted by the multiphoton ionization model. The validity of EID calculations was tested by comparing the results of comprehensive propagation simulations with data from experiments on the filamentation of terawatt picosecond CO_2_ laser pulses^55^. It was shown that the plasma electrons produced through the EID mechanism provide a necessary seed for avalanche ionization, which then produces enough electrons to stabilize the laser pulse propagation, leading to extremely long and wide filaments.

The apparent discrepancy between the experiments of Lai et al.^48^, showing the validity of the PPT ionization rate formula, and the EID calculations can be explained by the fact that the experiments by Lai et al. were conducted at low gas pressures, where the many-body interactions lose their importance. To clarify this situation, Woodbury et al.^56^ experimentally measured the ionization yield in air, nitrogen, and argon at atmospheric (0.5–3 bar) pressures for 1.024 and 3.9 μm picosecond laser pulses. The ionization yield was measured over a dynamic range of intensities covering 14 orders of magnitude (the sensitivity of the experiment allowed to observe individual ionization events at low intensities). The obtained experimental results can be summarized as follows. At low intensities (<4 TW/cm^2^), where the largest relative contribution from EID ionization is expected, the ionization yield was consistent with the multiphoton ionization rate of an unknown contaminant (found in all considered gases) with ionization energy *U*_i_ = 6 eV. Neither the wavelength-independent *I*^2^ scaling nor the wavelength-insensitive absolute yield suggested by EID was observed. In addition, the measured yields were a factor of 10^6^ lower than those predicted by EID. At higher intensities (>10 TW/cm^2^), the ionization yields were consistent with the multiphoton or tunneling ionization rates of isolated molecules calculated by the PPT formula^49^ with a generalized Coulomb prefactor^50^. At the moment, the source of the aforementioned contradiction between the experiments and theory remains unclear and requires further study.

Recent numerical simulations showed that seed electrons for avalanche ionization can come from the ionization of air aerosols^57^. Similar to the EID mechanism, this scenario can explain the appearance of additional free electrons (which otherwise cannot be produced through an avalanche seeded solely by tunnel ionization), which are necessary to obtain the long and stable far-infrared filaments observed in the experiment^55^.

In addition to the ionization yield measurements, Woodbury et al.^56^ estimated the effective collisional frequency *ν*_c_, which determines the avalanche ionization rate. *ν*_c_ = 0.55 ps^−1^ was found for both the 1.024 and 3.9 μm wavelengths. However, in view of the growing role of many-body effects in laser-plasma interactions with mid-infrared and far-infrared laser pulses, a recent theoretical study by Wright et al.^58^ put the simple, one-parameter description of the avalanche ionization rate into question. They proposed a two-temperature model, which provides a microscopically motivated foundation for avalanche ionization in gases with long-wavelength laser pulses. The basic idea behind this model is that free electrons, moving in the laser field after photoionization occurs, need some time to acquire a large enough kinetic energy to collisionally ionize neighboring atoms or molecules. As a consequence, avalanche ionization does not instantaneously follow the laser intensity, leading to memory or transient effects. In particular, the plasma density can continue to increase well after the pulse has passed due to the presence of high-energy electrons that store enough energy for impact ionization of the neutral atoms or molecules at later times.

The increasing role of free electrons in the overall nonlinear response of a medium can lead to even more exotic scenarios. For example, numerical simulations by Gao and Shim^59^ showed that a 15 μm laser pulse propagating in a weakly ionized argon gas can undergo self-focusing (at a power much lower than the critical power for Kerr self-focusing) due to the transverse variations in intensity-dependent electron-impact ionization.

From the above discussion, it becomes clear that one should be very careful in modeling the ionization and electron dynamics driven by mid-infrared and far-infrared fields. Electron densities and energy distributions sensitively depend on the driving field wavelength, and there are still a number of blind spots and controversies in our understanding of these dependences. However, the classical treatment of electron dynamics allowed at these frequencies can greatly simplify the theory. To date, the simulations of THz generation from mid-infrared filamentation account for the simplified theory and seem to reproduce the experimental findings well, as we will show in the following.

### Nonlinear polarization: Kerr and Raman effects

The effect of self-focusing lies in the basis of laser filamentation and, alongside ionization, is responsible for the formation of the extended plasma channels necessary for effective THz generation. This nonlinear effect leads to an intensity-dependent change in the total refractive index *n* = *n*_0_ + *n*_2_*I*, where *n*_0_ is the linear part of the refractive index, *n*_2_ is the nonlinear index, and *I* is the laser intensity. In molecular gases, such as nitrogen or oxygen, there are two contributions to the nonlinear index *n*_2_. The first one (Kerr) is nearly instantaneous and originates from the nonlinear response of bound electrons to the external electric field. The second one (Raman) is time-dependent and arises from the tendency of gas molecules to align themselves along the field polarization. Due to inertia during the rotation of molecules, the molecular alignment lags behind the driving field and results in a delayed increase in polarizability, which, in turn, leads to an increase in the refractive index near the trailing end of the laser pulse. The combination of these two effects gives rise to a nonlinear polarization, *P*_nl_, which can be modeled as

4 $$P_{{\mathrm{nl}}}(r,t) = {\varepsilon _0}\chi ^{(3)}{\left[ (1 - f_{\mathrm{R}})E^2(r,t) \,+\, f_{\mathrm{R}} {\int \nolimits_{-\infty }^t} {R(t - t^{\prime})} E^{2}(r,t^{\prime})dt^{\prime}\right]}E(r,t)$$

Here, *χ*^(3)^ is the total (Kerr plus Raman) third-order susceptibility, and $f_{\mathrm{R}} \in [0,1]$ is the fraction of the Raman contribution. For subpicosecond laser pulses, the response function *R*(*t*) can be calculated based on the damped harmonic oscillator model^60,61^:

5 $$R(t) = R_0{\mathrm{exp}}( - {\mathrm{{\Gamma}}}t/2){\mathrm{sin}}({\mathrm{{\Lambda}}}t)$$

where $R_0 = ({\mathrm{{\Gamma}}}^2/4 + {\mathrm{{\Lambda}}}^2){\mathrm{{\Lambda}}}^{ - 1}$ is a normalization factor and Γ and Λ are the damping and oscillation frequencies, respectively.

The total nonlinear index *n*_2_ linearly depends on *χ*^(3)^ and can be written as $n_2 = 3\chi ^{(3)}(4\varepsilon _0n_0^2c_0)^{ - 1}$. We can express *n*_2_ as a sum of nonlinear indices *n*_2K_ and *n*_2R_, responsible for, respectively, the Kerr and Raman contributions: $n_2 = n_{2{\mathrm{K}}} + n_{2{\mathrm{R}}}$, where $n_{2{\mathrm{K}}} = (1 - f_{\mathrm{R}})n_2$ and $n_{2{\mathrm{R}}} = f_{\mathrm{R}}n_2$.

**Fig. 5:**
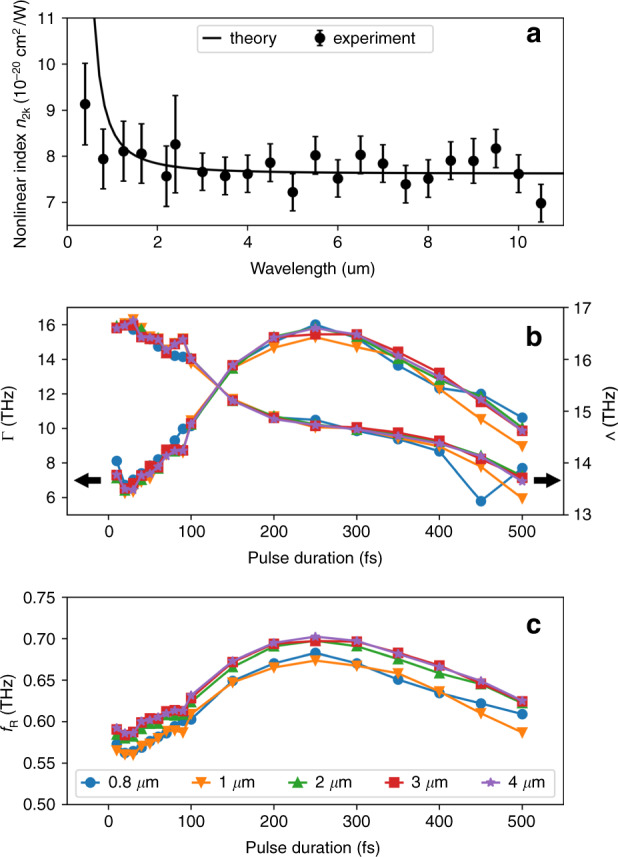
Parameters of Kerr and Raman contributions to the nonlinear index of air. **a** Dependence of the Kerr nonlinear index for air on the laser wavelength: experimental data obtained by Zahedpour et al.^62,63^ (points) and theory developed by Brown et al.^64^ according to Eq. (6) (line). **b** Values of frequencies Γ and Λ determining the Raman response function *R*(*t*) (arrows indicate the associated scale), and **c** the fraction of the Raman contribution *f*_R_ in air as a function of the pulse duration for wavelengths of 0.8–4 μm. Figures **b** and **c** are reprinted from^61^ with permission from the American Physical Society

Since most of the experiments on two-color filamentation, due to their simplicity, are conducted in air, let us consider the nonlinear indices of air in more detail. While the values of the Kerr nonlinear index *n*_2K_ for air have been measured for various wavelengths, from the ultraviolet up to the near-infrared parts of the spectrum, until recently, there were no corresponding data for the mid-infrared and far-infrared spectral range. Currently, the most comprehensive measurements of *n*_2K_ for different air constituents were made by Zahedpour et al.^62,63^ using the single-shot supercontinuum spectral interferometry technique. They presented the experimental values of the Kerr nonlinear index for nitrogen and oxygen in a very wide range of wavelengths from 0.4 to 11 μm. With these data, we can calculate the values of *n*_2K_ for air as a weighted sum $n_{2{\mathrm{K}}} = 0.79n_{2{\mathrm{K}}}^{(N_2)} + 0.21n_{2{\mathrm{K}}}^{(O_2)}$, where $n_{2{\mathrm{K}}}^{(N_2)}$ and $n_{2{\mathrm{K}}}^{(O_2)}$ are the Kerr nonlinear indices of nitrogen and oxygen, while the factors 0.79 and 0.21 are the typical fractions of these gases in air. Figure 5a shows the resulting wavelength dependence of the Kerr nonlinear index *n*_2K_ for air. Evidently, despite some noise, the obtained values of *n*_2K_ demonstrate that over the studied range of wavelengths, the Kerr nonlinear response of air is quite dispersionless.

In turn, Brown et al.^64^ used ab initio calculations based on the time-dependent density functional theory to compute the Kerr nonlinear index *n*_2K_ for nitrogen and oxygen at near-infrared and mid-infrared wavelengths in the range from 1 to 4 μm. It was shown that the calculated values of *n*_2K_ can be well fitted by a Sellmeier-like equation:

6 $$n_{2{\mathrm{K}}}(\lambda ) = \frac{{P^{ - 1}}}{{\lambda _0^{ - 2} - \lambda ^{ - 2}}}$$

where *P* = 14.63 GW, *λ*_0_ = 0.3334 μm for nitrogen and *P* = 14.62 GW, *λ*_0_ = 0.3360 μm for oxygen, with the wavelength *λ* measured in μm (as before, the nonlinear index for air can be calculated as the corresponding weighted sum). Figure 5a shows that Eq. (6) provides a very good fit for the experimental data obtained by Zahedpour et al.^62,63^ and confirms the flat and featureless landscape of air *n*_2K_ dispersion at mid-infrared and far-infrared wavelengths.

Additionally, Brown et al.^64^ found that over the wavelength range of 2–4 μm, the Kerr nonlinear index *n*_2K_ of air is insensitive to the laser intensity up to intensities as high as 10^14^ W/cm^2^ (though at shorter wavelengths, a weak intensity dependence, caused by nonnegligible ionization, was observed for intensities above 5 × 10^13^ W/cm^2^). These findings are in line with recent experiments that showed that for near-infrared laser pulses, the nonlinear response of bound electrons preserves its linear dependence on intensity even in the presence of substantial ionization, well beyond the limits of perturbation theory^65^. In turn, any dependence of the Kerr nonlinear index *n*_2K_ on intensity would signal that there are some nonlinearities of higher order that we do not take into account. Since with increasing wavelength, ionization tends to occur in the tunnel regime and thus becomes independent of the wavelength, one can assume that the above conclusions on the independence of *n*_2K_ from the intensity can be safely extended to laser pulses with even longer wavelengths located further in the far-infrared spectral region. Thus, we can conclude that in the range of wavelengths from near-infrared to far-infrared and at intensities up to 10^14^ W/cm^2^, the dominant contribution to the nonlinear polarization comes from the effects of the third order, while higher orders of nonlinearity can be neglected.

In addition to the *n*_2K_ values, Zahedpour et al.^62,63^ also measured the Raman nonlinear index *n*_2R_ for nitrogen and oxygen in the same wavelength range from 0.4 to 11 μm. According to the obtained results, the value of *n*_2R_ for air (calculated through the corresponding weighted sum) is constant over the considered wavelength range and equal to approximately 3 × 10^−20^ cm^2^/W. Taking into account the values of the Kerr nonlinear index *n*_2K_ presented in Fig. 5a, this *n*_2R_ value suggests a fraction of the Raman contribution $f_{\mathrm{R}} = n_{2{\mathrm{R}}}/(n_{2{\mathrm{K}}} + n_{2{\mathrm{R}}}) \approx 0.8$.

In parallel, Langevin et al.^61^ numerically studied the molecular Raman contribution in air at mid-infrared wavelengths. By solving the time-dependent Schrödinger equation, they calculated the rotational molecular response of air for laser pulses with wavelengths from 0.8 to 4 *μ*m, pulse durations from 10 to 500 fs, and intensities from 0.2 to 20 TW/cm. It was demonstrated that the treatment of the Raman contribution by Eq. (4) with the damped harmonic oscillator response function given by Eq. (5) can be safely extended to mid-infrared wavelengths. Figure 5b shows the two frequencies, Γ and Λ, calculated by Langevin et al.^61^ for air in the range of different wavelengths and pulse durations. According to this figure, the values of Γ and Λ are almost independent of the wavelength but exhibit a moderate dependence on the pulse duration. In turn, Fig. 5c shows that, depending on the pulse duration, the value of Raman fraction *f*_R_ smoothly changes in the range from 0.55 to 0.7 but, similar to the characteristic frequencies, remains nearly independent of the wavelength.

From Eq. (5), we find that the peak of the response function *R*(*t*) occurs at time $t_{\mathrm{p}} = {\mathrm{{\Lambda}}}^{ - 1}{\mathrm{tan}}^{ - 1}(2{\mathrm{{\Lambda}}}/{\mathrm{{\Gamma}}})$. According to the values of Γ and Λ calculated by Langevin et al.^61^, *t*_p_ lies in the range between 75 and 90 fs, which roughly indicates the pulse durations above which the Raman contribution will have a significant impact on the refractive index change. Therefore, taking into account the above values of *f*_R_, we can conclude that for laser pulses with durations above approximately 75 fs, the molecular Raman contribution prevails over the electronic Kerr response for all wavelengths from the near-infrared to the mid-infrared region.

In addition, numerical simulations of laser filamentation in air performed by Langevin et al.^61^ demonstrated that the Raman contribution manifests itself in exactly the same way (through a considerable red-shift of the laser spectrum) for all near-infrared and mid-infrared laser pulses.

We also mention the experiments by Pigeon et al.^66,67^, where the total nonlinear index of air *n*_2_ = 50 × 10^−20^ cm^2^/W was measured for 200 ps, 10.6 μm laser pulses generated by a pulsed CO_2_ laser. However, due to the long pulse duration and the time-integrated measurements, the Kerr and Raman contributions in these experiments cannot be separated.

In summary, the above results show that the nonlinear polarization response of air at mid-infrared and far-infrared wavelengths is explored in depth, both experimentally and theoretically. The good quantitative agreement between the theory and experimental data indicates that the basic underlying physics is well understood, while the obtained values of the Kerr and Raman response parameters can be used for comprehensive simulations of THz generation by mid-infrared and far-infrared two-color filamentation.

### Dispersion of air refractive index

Due to its simplicity, most of the experiments on THz generation by two-color filamentation are conducted in air. By itself, air is a mixture of different gases, including nitrogen N_2_, oxygen O_2_, water vapor H_2_O, carbon dioxide CO_2_, and several others. Depending on its wavelength, electromagnetic radiation propagating in air can fall in resonance with various electronic transitions, as well as vibrations and rotations in the above molecular gases, which will result in the absorption of this radiation. As a result, only radiation of specific wavelengths, lying in so-called transparency windows, can propagate in air without losses. Therefore, before developing any specific laser system for two-color filamentation in air, it is necessary to ensure that the wavelengths of its fundamental and second harmonic fall into one of these transparency windows. This is especially important for laser pulses from mid-infrared and far-infrared parts of the spectrum, which are densely populated by absorption lines corresponding to the vibrations and rotations of H_2_O and CO_2_ molecules.

**Fig. 6:**
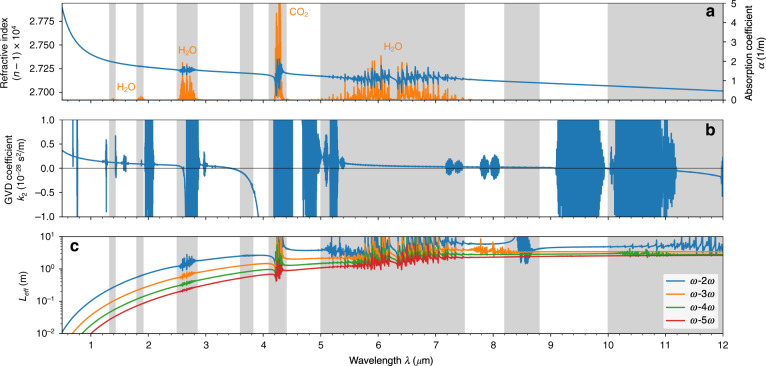
Dispersion of air. Wavelength dependence of **a** the refractive index *n* (blue) and absorption coefficient *α* (orange), **b** the group velocity dispersion coefficient *k*_2_, and (**c**) the phase velocity walk-off length *L*_eff_ for several pairs of frequencies

Figure 6a shows the real part of the air refractive index $n(w)$, as well as the corresponding absorption coefficient $\alpha(w)$ calculated using the HITRAN database^68,69^ for wavelengths in the range from 0.5 to 12 μm. We can see that the presence of H_2_O molecules in air gives rise to several groups of absorption lines between 1.3 and 3 μm, as well as to a wide absorption band located between 5 and 7.5 μm. In turn, the presence of CO_2_ molecules leads to the strong absorption of radiation at wavelengths from 4.1 to 4.4 μm. With this knowledge of air absorption bands, we can determine the wavelengths of possible laser sources suitable for two-color filamentation. The shaded areas in Fig. 6 indicate the spectral regions where the central wavelength of either the fundamental pulse or its second harmonic falls into one of the absorption bands. As we can see, the choice of allowed wavelengths in the mid-infrared and far-infrared part of the spectrum is quite restricted.

One should also be aware of another phenomenon that can potentially shrink the selection of possible wavelengths even further. Filamentation of ultrashort laser pulses is accompanied by broadening of their spectra and the generation of a supercontinuum. Therefore, if the central wavelength of a laser pulse is located close to one of the absorption bands, a part of the nonlinearly expanding spectrum will penetrate the region of resonance frequencies and will be continuously absorbed^69,70^. Under certain conditions and especially under loose focusing, this nonlinearly enhanced, through spectral broadening, linear absorption can dominate other sources of losses and strongly alter the process of filamentation and generation of plasma^69^.

Among all of the air transparency windows, the one from 3.8 to 4.1 μm draws special attention. As shown in Fig. 6b, this spectral range is characterized by large negative values of the group velocity dispersion coefficient $k_2 = \partial ^2k/\partial \omega ^2$, which corresponds to anomalous group velocity dispersion. The propagation of ultrashort laser pulses in anomalously dispersive nonlinear media is characterized by temporal self-compression and the formation of quasi-solitonic light bullets that lead to the formation of more uniform, elongated plasma channels, thus providing more favorable conditions for THz generation.

One of the essential prerequisites for efficient THz generation by two-color filamentation is the properly chosen phase difference between the fundamental and second harmonic pulses, which guarantees optimal field symmetry breaking and thus maximizes the photocurrent oscillating at THz frequencies (see Fig. 2). However, since the air refractive index varies with frequency, the fundamental and second harmonic waves propagate at different phase velocities. As a result, the optimal THz generation phase difference can be sustained over only a limited propagation length. We can estimate this length as $L_{{\mathrm{eff}}} = (\lambda /2)/|n(\omega ) - n(2\omega )|$, where $\lambda = 2\pi c_0/\omega$ is the central wavelength of the fundamental wave and *n*(*ω*) and *n*(2*ω*) are the linear refractive indices at a given frequency. The effective length *L*_eff_ corresponds to the distance where the phase difference between the fundamental and second harmonic waves changes by *π* and indicates the maximum length of effective THz generation. Figure 6c shows the dependence of *L*_eff_ on the wavelength *λ*. It is clearly seen that during the transition from the near-infrared to the mid-infrared spectral range, the effective length *L*_eff_ rapidly increases until it saturates in the far infrared region. For comparison, for laser wavelengths of 0.8, 3.9, and 10 μm, *L*_eff_ is equal to 5 cm, 2.6 m, and 4.9 m, respectively. Thus, by using laser pulses with longer wavelengths, it is possible to maintain the optimal phase difference between the fundamental and second harmonic pulses over a longer propagation distance, thereby increasing the length of efficient THz generation.

Beyond the phase difference discussed above and its implications, the difference in group velocities of the fundamental and second harmonic pulses leads to a temporal walk-off between the two-color pulses along the propagation. This temporal walk-off for a given distance *z* can be calculated as: $\triangle{t}_g=[v^{-1}_g(w)-v^{-1}_g(2w)]z$, where $v_g(w)$ and $v_g(2w)$ are the group velocities of the fundamental wave and its second harmonic, respectively. For example, after one meter of propagation in air of fundamental pulses with central wavelengths of 0.8, 3.9, and 10 μm, their second harmonics will lag behind by, respectively, 81, 0.15, and 2.2 fs. Therefore, we find that the group velocity walk-off reduces in the mid-infrared and far-infrared spectral regions, thereby making preferable the choice of longer driving wavelengths for two-color filamentation.

Apart from the effective length $L_{\mathrm{eff}}$ the waves at fundamental and second harmonic frequencies (*ω* – 2*ω*), Fig. 6c also shows *L*_eff_ for several other harmonics (*ω*–3*ω*, *ω*–4*ω*, and *ω*–5*ω*) appearing during two-color filamentation as a part of the generated supercontinuum. Similar to the case of the second harmonic, the corresponding values of *L*_eff_ increase with wavelength. In other words, the higher harmonics of long-wavelength laser pulses remain in phase with the wave at the fundamental frequency over a considerable propagation distance. As a result of this continuous phase locking, the underlying optical carrier wave exhibits extreme shock formation, as in the case of an ideal nondispersive medium^71^. Under certain conditions, in the case of collimated or weakly focused laser beams, this shock wave formation may occur well before ionization can generate a sufficient amount of plasma to stop the intensity growth caused by Kerr self-focusing^72^. In this situation, shock wave formation becomes a dominant mechanism of intensity clamping, which may no longer require the generation of plasma. Under these loose focusing conditions and in the absence of a sufficient number of free electrons, one can expect the suppression of THz generation.

A recent theoretical study predicts another unusual nonlinear-optical effect caused by anomalous dispersion of the air refractive index^73^. It was shown that nonlinear propagation of a long-wavelength ultrashort laser pulse in a medium with a broad and weak region of anomalous dispersion (as an example, the authors considered 100 fs pulses at a 10 μm wavelength) can be accompanied by the formation of attosecond (≈0.66 fs) subcycle ripples on the temporal pulse profile. It was predicted that these ripples can occur during the initial stage of propagation, well before the self-focusing collapse point. The above results echo earlier theoretical studies that predicted the formation of intense attosecond shock waves for near-infrared laser pulses^74^. In general, the appearance of extremely short sub-cycle formations with high intensity can locally enhance the yield of tunneling electrons and thus alter their acceleration and deceleration phases in a laser field. As a result, one can expect a strong disturbance of the optimal phase between the fundamental and second harmonic waves and thereby disruption of the process of THz generation.

In summary, the linear medium absorption places strict limits on the operational spectral windows where nonlinear propagation can be effectively studied, while anomalous dispersion regions can be useful for solitonic-like propagation.

In the following, we use the microscopic description of light-matter interactions with intense mid-infrared and far-infrared light fields, discussed in detail in the last three sections, to explore the generation of THz radiation from mid-infrared and far-infrared filaments.

## THz generation

Early evidence of stronger THz radiation at mid-infrared wavelengths was obtained from particle-in-cell (PIC) simulations of 4 μm single-color laser pulses interacting with a hydrogen gas target^75^. It was shown that the amplitude of the emitted THz pulses is enhanced by 35 times as the laser wavelength increases from 1 to 4 μm. However, since the single-color pulses cannot provide effective field symmetry breaking and PIC simulations do not take into account propagation effects, it was difficult to extrapolate these results to the case of actual experiments on two-color filamentation.

In a later study, a comprehensive model based on the unidirectional pulse propagation equation (UPPE) was used to simulate THz generation by two-color laser filamentation in argon^76^. Among other things, the authors compared two-color filamentation with 0.8 and 2 μm laser pulses and showed that, in the latter case, the THz energy is approximately ten times higher.

**Fig. 7:**
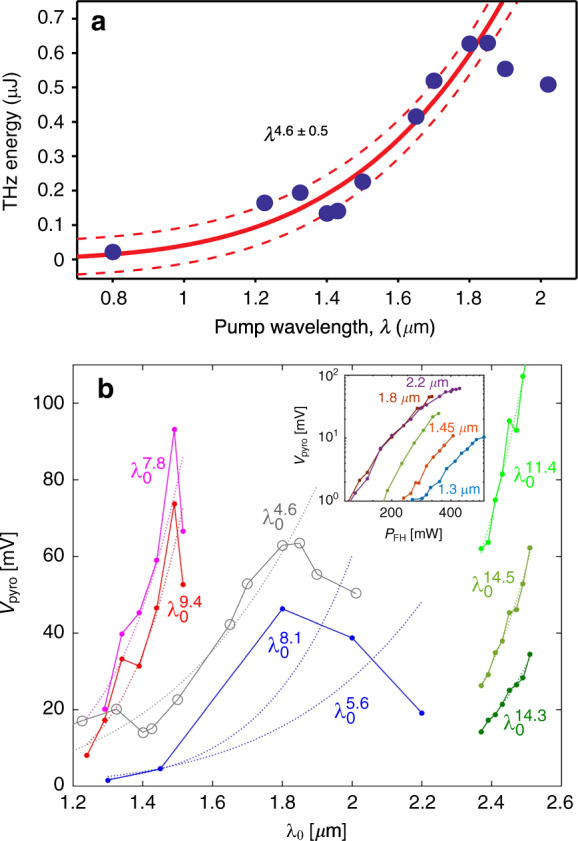
Scaling of THz energy with the laser wavelength. **a** THz energy measured by Clerici et al.^77^ for 12 different pump wavelengths (solid circles). The red solid curve shows the power law fit *λ*^4.6±0.5^ together with the 65% confidence bounds (red dashed curves). **b** Wavelength dependence of the pyroelectric detector signal indicating the THz energy yield in the experiments by Nguyen et al.^78^. Different point sets correspond to different OPAs and input pulse energies. The dotted curves provide the *λ*^*a*^ fitting. The gray circles correspond to the data obtained by Clerici et al.^77^. Figure **a** is reprinted from ref. ^77^ with permission from the American Physical Society, and **b**, from ref. ^78^ with permission from the Optical Society of America

The first experimental study of THz generation by two-color filamentation at different wavelengths, in the range from 0.8 to 2.02 μm, was carried out by Clerici et al.^77^. The obtained results, reproduced in Fig. 7a, clearly show a fast increase in the THz energy with increasing laser wavelength *λ*. The growth of the THz yield extended up to a wavelength of 1.85 μm, where the THz energy reached its maximum value of approximately 0.65 μJ, which is approximately 30 times larger than that at 0.8 μm. The strength of the corresponding THz field was estimated to be 4.4 MV/cm. However, at wavelengths above 1.85 μm, the observed THz energy unexpectedly decreased. In addition, the initial increase in the THz yield could be well fitted by a *λ*^4.6^ power law, while the photocurrent model predicts at most a *λ*^2^ dependence. The authors partially justified the observed deviations from the theory by their specific experimental conditions, such as the focusing and input energy limitations. Another suggested idea was that the length and radius of the generated plasma channels depend on the laser wavelength, which introduces a correction to the *λ*^2^ scaling and could explain the saturation of THz energy at longer wavelengths.

Subsequent theoretical studies questioned the very possibility of describing the wavelength-dependent scaling of the THz yield as a simple *λ*^*a*^ power law with a universal power *α*^79^. For the example of two-color laser pulses with wavelengths from 0.8 to 2 μm, it was demonstrated that depending on the relative phase between the *ω* and 2*ω* pulse components, as well as their envelopes, the photocurrent model can justify powers *a* between 2 and 5. In turn, UPPE simulations of two-color filamentation performed in propagation geometry demonstrated *λ*^*a*^ scaling of the THz energy yield with a power *a* ranging from 2 to 3.5.

The anomalous growth in THz energy with laser wavelength was specifically addressed in recent experiments by Nguyen et al.^78^, where two distinct optical parametric amplifiers (OPAs) were used to generate two-color laser pulses with fundamental wavelengths from 1.2 to 2.6 μm. As a result, THz energies were measured to scale like *λ*^*a*^ with large exponents *a* ranging from 5.6 to 14.3 (see Fig. 7b). By means of comprehensive 3D simulations, Nguyen et al. demonstrated that these high scaling powers can be caused by variations in the laser beam size and pulse duration during the process of tuning the OPA carrier wavelength, as well as by subsequent deviations of the phase-matching conditions during the generation of the second harmonic component. In particular, it was shown that the lack of control of the phase difference between the *ω* and 2*ω* laser pulse components can explain the saturation of the THz energy yield at longer wavelengths.

We also would like to mention the experiments by Zhao et al.^80^, who studied two-color filamentation in different gases (helium, neon, argon, nitrogen, krypton, and xenon) at different wavelengths from 1.2 to 1.6 μm. The THz energy in these experiments was measured as a function of the input pulse energy, gas species, gas pressure, and pump wavelength. As a result, it was shown that stronger terahertz signals are more likely to be produced by longer wavelengths in heavier gases.

**Fig. 8:**
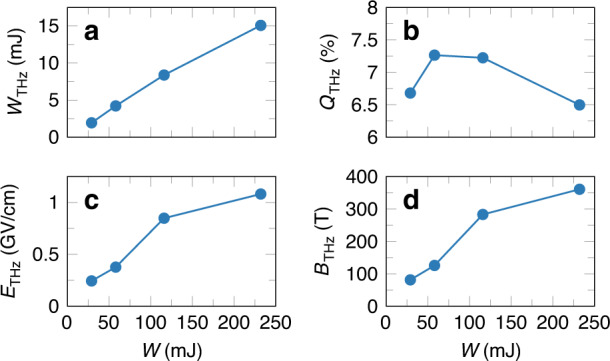
Various parameters of THz radiation generated by mid-infrared filaments. **a** THz pulse energy *W*_THz_, **b** THz conversion efficiency *Q*_THz_, **c** estimated peak electric *E*_THz_, and **d** magnetic field *B*_THz_ of a focused THz pulse versus the input energy *W* of 3.9 μm two-color laser pulses. Reprinted from ref. ^81^ with permission from the American Physical Society

The above studies of two-color filamentation were carried out using typical OPAs as a source of the long-wavelength laser pulses. Due to the restrictions of the OPA technique, the peak power of the obtained mid-infrared laser pulses was less than the critical power *P*_cr_ for Kerr self-focusing, thereby far from optimal for filamentation. Taking into account the appearance of new OPCPA laser systems capable of generating 3.9 μm mid-infrared laser pulses with a peak power exceeding *P*_cr_, the THz generation by two-color filamentation in air at 3.9 μm was compared to the case of 0.8 μm in recent numerical studies by the present authors^81,82^. Figure 8 shows a brief summary of the obtained results. According to these results, with 3.9 μm two-color laser pulses, one can achieve a THz conversion efficiency close to 7% (see Fig. 8b), which is more than two orders of magnitude higher than the 0.8 μm case and appears to be the highest THz conversion efficiency reported to date compared to any of the known approaches for THz generation. It was shown that by further scaling of the input laser energy, it would be possible to generate multi-millijoule THz pulses (see Fig. 8a) with electric and magnetic fields at the GV/cm and kT levels, respectively (see Fig. 8c, d). The high conversion efficiency of THz radiation driven by 3.9 μm two-color laser pulses, in comparison with the near-infrared ones, was explained by the synergy of the following factors: stronger photocurrents caused by higher ponderomotive forces, negligible walk-offs between harmonics, and longer and wider plasma channels. In addition, the authors predicted the existence of a novel mechanism in which higher harmonics generated in the process of two-color filamentation contribute to field symmetry breaking and thus enhance THz generation. The impressive increase in the THz conversion efficiency using 3.9 μm two-color laser pulses in air was later confirmed by additional numerical simulations performed by Nguyen et al.^83^, who also considered the case of two-color filamentation at 10.6 μm.

**Fig. 9:**
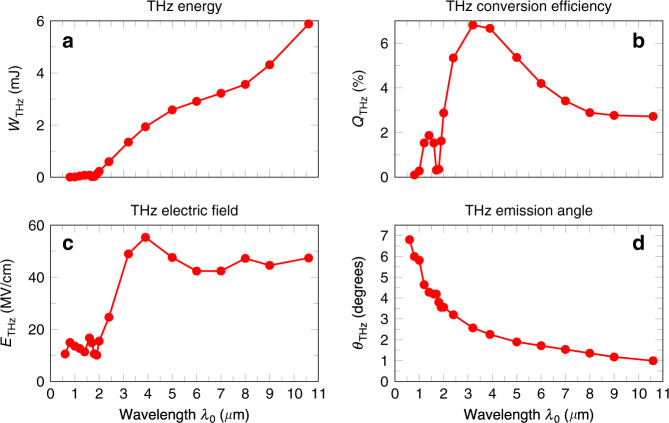
Dependence of various THz parameters on the laser wavelength. **a** Energy of the generated THz pulse *W*_THz_, **b** THz conversion efficiency *Q*_THz_, **c** peak THz electric field *E*_THz_, and **d** THz emission angle *θ*_THz_ versus the wavelength *λ*_0_ of the fundamental laser pulse. Reprinted from^84^ with permission from the Optical Society of America

To find an optimal laser wavelength for two-color filamentation in air, the whole range of possible two-color laser sources with fundamental wavelengths from 0.6 to 10.6 μm was considered in a series of full 3D numerical simulations by the present authors^84^. For a fair comparison, the ratio of the laser pulse peak power to the critical power of self-focusing in these simulations was fixed at 1.2, independent of the wavelength. As a brief outcome of the obtained results, Fig. 9 shows how different parameters of the emitted THz radiation change with the wavelength. First, one can see that the THz energy grows non-monotonically with the wavelength, albeit without saturation (see Fig. 9a). Next, Fig. 9b shows that the THz conversion efficiency *Q*_THz_ depends on the wavelength *λ* in a quite peculiar way, increasing and decreasing at wavelengths below 1.8 μm, then reaching the global maximum of approximately 7% at approximately 3.2 μm, and finally decreasing towards far-infrared wavelengths. The presence of the global maximum near *λ* = 3.2 μm allows one to conclude that this laser wavelength is the optimal one for all THz sources based on two-color filamentation in air. In turn, Fig. 9c demonstrates that around *λ* = 4 μm, the initial growth of the THz field strength saturates at approximately 50 MV/cm within the filament. For applications, one can collect this THz radiation after the filament and refocus it, achieving electric field strengths at the GV/cm level^81,82^. Finally, Fig. 9d shows that the THz conical emission angle monotonically decreases with the wavelength, making the THz radiation from mid-infrared and far-infrared laser pulses better directed and easier to collect. As explained by the interference model^85–87^, this effect is linked to the longer and thicker filament plasma channels generated by laser pulses with longer wavelengths.

The first experimental evidence of THz generation by 3.9 μm two-color laser pulses was presented at the CLEO conference in 2018^88^. In these experiments, the authors used the high-power 3.9 μm OPCPA laser system developed at the TU Wien Photonics Institute^32^. However, due to the unoptimized experimental conditions, the maximum achieved THz energy at that time was only ~49 μJ, with the corresponding THz conversion efficiency being ~0.77%, which nonetheless was almost two orders of magnitude higher compared to the typical values reported for 0.8 μm two-color laser pulses.

At the 2019 CLEO conference, the same group of authors presented the results of THz generation by 3.9 μm two-color laser pulses obtained in a better optimized experiment on two-color filamentation^89^. Over the year, the energy of the emitted THz pulses increased up to 0.185 mJ, and the THz conversion efficiency increased up to 2.36%. The details of these experiments can be found in^90^.

**Fig. 10:**
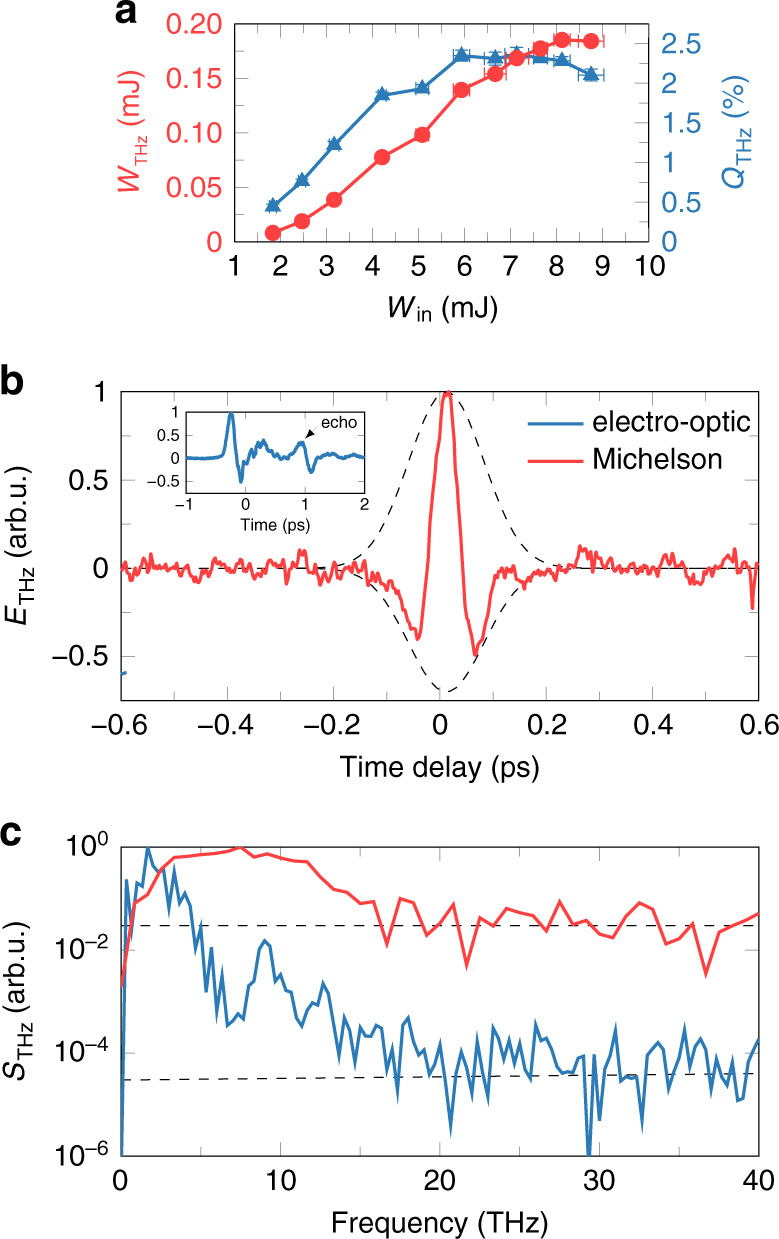
Energy, temporal, and spectral characteristics of THz pulses generated by 3.9 *μ*m filaments. **a** Dependence of THz energy *W*_THz_ and the corresponding THz conversion efficiency *Q*_THz_ on the input laser energy *W*_in_. **b** THz signals *E*_THz_ measured by electro-optic sampling (inset, blue) and by a Michelson interferometer (red). **c** THz power spectra *S*_THz_ (solid lines) obtained by the above techniques together with the noise levels of each technique (dashed lines). Reprinted from ref. ^90^ under the terms of the Creative Commons CC BY license

Figure 10 presents various parameters of the obtained THz pulses. In particular, Fig. 10a shows that with increasing input laser energy *W*_in_, the energy of the THz pulses grows and reaches a maximum value of 0.185 mJ for *W*_in_ equal to 8.12 mJ. In turn, the corresponding maximum of the THz conversion efficiency reaches 2.36%. At the same time, the measurements based on both electro-optic sampling and Michelson interferometry demonstrate that these high-energy THz pulses are single-cycle pulses with femtosecond duration and a spectral width of at least 20 THz (see Fig. 10b, c). At this moment, the above values of the THz energy and THz conversion efficiency remain the highest among all values previously reported for plasma-based THz sources. Moreover, according to numerical simulations benchmarked by the experimental data, by further optimization of the existing experimental setup, it would be possible to significantly upscale the observed THz yield and reach a THz conversion efficiency of at least ~5%^90^.

Since then, two more experiments on two-color filamentation have demonstrated the significant enhancement of the THz yield for 3.9 μm laser pulses^91,92^. In both of them, THz radiation was studied as a part of the broadband supercontinuum generated by 3.9 μm two-color laser pulses jointly with the supercontinuum of higher harmonics. In the first experiment, conducted by Jang et al.^91^, the observed THz conversion efficiency was ~1%, with the corresponding THz energy being close to 30 μJ. In addition, Jang et al.^91^ studied the dependence of the energy of THz pulses generated by 3.9 μm laser pulses on the initial *ω* − 2*ω* phase difference and found that it has the same *π*/2 period as in the case of near-infrared laser pulses.

In the second experiment, carried out by Mitrofanov et al.^92^, THz generation by 3.9 μm two-color laser pulses was studied in various gases (helium, argon, nitrogen, air, and krypton) under different pressures. Gases with a lower ionization potential were shown to provide a higher THz yield. In particular, under the fixed energy of the 3.9 μm laser pulse, the highest THz energy (24 μJ) was achieved in the experiments with krypton. For comparison, the highest THz energy measured in air was 18 μJ (corresponding to a 5 MV/cm THz field after focusing).

In Table 1, we summarize the parameters of the THz pulses generated in experiments using two-color filamentation with different laser wavelengths. The existing mid-infrared laser sources are clearly able to generate THz fields with energies and amplitudes exceeding, respectively, 0.1 mJ and 0.1 GV/cm. According to the theoretical predictions, in the near future, we can expect the appearance of THz sources capable of generating multi-millijoule THz pulses with peak THz electric fields at the GV/cm level. To gain some intuition about the strength of these THz fields, let us consider them in the context of current THz-based electron accelerators. Since the electron energy gain and acceleration gradient scale linearly with the THz field amplitude, one would expect GV/cm THz fields to allow achieving multi-GeV/m gradients and electron energy gains exceeding 10 MeV—numbers that are characteristic of electron accelerators based on petawatt laser technologies^7,8^.

**Table 1:** Energy *W*_THz_, conversion efficiency *Q*_THz_, and peak electric field *E*_THz_ of THz pulses generated in experiments using two-color filamentation with different laser wavelengths *λ*

*λ* (µm)	*W*_THz_ (µJ)	*Q*_THz_ (%)	*E*_THz_ Ref. (MV/cm)
0.8	1.44	0.01	8^17^
0.8	31	0.07	21^18^
1.5	0.099	0.56	–^80^
1.85	0.63	0.16	4.4^77^
3.9	18	0.3	5^92^
3.9	27	1	–^91^
3.9	185	2.36	150^90^

## Conclusions

In summary, we have reviewed the most promising way to date of developing high-peak-power THz sources. THz sources driven by ultrashort mid-infrared and far-infrared two-color laser filamentation demonstrate unprecedented conversion efficiencies on the level of 5–7%. Starting from the fundamental microscopic physical mechanisms, we drove all the way to nonlinear propagation in the form of filaments and explained the physics behind these sources. We showed that although some aspects of the fundamental processes have still not been fully elucidated, such as the role of many-body effects, quantitative agreement between the theory and the experiments can be achieved if one accounts carefully for the complex spatiotemporal phenomena of two-color filamentation.

The development of powerful THz sources similar to the ones we discussed here opens up new horizons in THz nonlinear science. The experimental findings and the most recent numerical simulations we discussed here point to a new generation of extreme-power THz transients. Near-future THz sources, driven by two-color mid-infrared and far-infrared filaments, are projected to offer multi-millijoule energies per pulse and peak electric and magnetic fields at the gigavolt per centimeter and kilotesla levels, respectively. Quasi-static ultrashort electric and magnetic bursts at these intensities will enable free space extreme nonlinear and relativistic science for not only accessing unexplored basic science problems but also offering unique solutions to demanding applications, such as compact charged particle accelerators.
